# Phylogenetic analyses and in-seedling expression of ammonium and nitrate transporters in wheat

**DOI:** 10.1038/s41598-018-25430-8

**Published:** 2018-05-04

**Authors:** Prabin Bajgain, Blake Russell, Mohsen Mohammadi

**Affiliations:** 1 Department of Agronomy, Purdue University, West Lafayette, IN 47907 USA; 2 Department of Agronomy and Plant Genetics, University of Minnesota, St. Paul, MN 55108 USA

## Abstract

Plants deploy several ammonium transporter (AMT) and nitrate transporter (NRT) genes to acquire NH_4_^+^ and NO_3_^−^ from the soil into the roots and then transport them to other plant organs. Coding sequences of wheat genes obtained from ENSEMBL were aligned to known AMT and NRT sequences of *Arabidopsis*, barley, maize, rice, and wheat to retrieve homologous genes. Bayesian phylogenetic relationships among these genes showed distinct classification of sequences with significant homology to *NRT*1, *NRT2*, and *NRT3* (*NAR2*). Inter-species gene duplication analysis showed that eight AMT and 77 NRT genes were orthologous to the AMT and NRT genes of aforementioned plant species. Expression patterns of these genes were studied via whole transcriptome sequencing of 21-day old seedlings of five spring wheat lines. Eight AMT and 52 NRT genes were differentially expressed between root and shoot; and 131 genes did not express neither in root nor in shoot of 21-day old seedlings. Homeologous genes in the A, B, and D genomes, characterized by high sequence homology, revealed that their counterparts exhibited different expression patterns. This complement and evolutionary relationship of wheat AMT and NRT genes is expected to help in development of wheat germplasm with increased efficiency in nitrogen uptake and usage.

## Introduction

Nitrogen (N) is a major element in plant physiology and metabolic processes. It is used in synthesis of amino acids, proteins^1^, and secondary metabolites^2^ as well as in signaling of several cellular and morphological processes that regulate plant growth and development^3^. Furthermore, nitrate, the primary source of inorganic nitrogen for plants, is involved in early growth of lateral roots^4^ and controls the ratio of root: shoot growth^5^.

N is available to the plant via uptake of two main molecules: ammonium (NH_4_^+^) and nitrate (NO_3_^−^). Plants have evolved to develop different affinity transport systems to cope with and operate under variable (high and low) external nitrogen availability^6,7^. These two uptake systems are referred to as low affinity transport system (LATS) and high affinity transport system (HATS)^8^. LATS is involved in transporting NO_3_^−^ at high external NO_3_^−^ concentrations (>1.1 mM)^9^. In contrast, HATS is activated when NO_3_^−^ is limited (<1.1 mM)^6^. The two main nitrate transporter (NRT) gene families are the low-affinity *NRT1* and the high-affinity *NRT2*^10^. The *NRT1* family is well documented and averages 54 family members in land plants^11^ of which only *NRT1.1*–*1.8* have been postulated to date^9^. The *NRT2* gene family is thoroughly characterized and consists of 7 members in *Arabidopsis*^12^. Similar to the NRT family, the ammonium transporter (AMT) genes are divided into *AMT1* and *AMT2* based on affinity levels. *AMT1* family consists of 1–7 members and *AMT2* family consists of 1–10 members^11^. For example, six AMT genes have been identified in *Arabidopsis* and ten in rice^13^. In contrast to NO_3_^−^, the HATS and LATS systems are different for NH_4_^+^ uptake^8^ as NH_4_^+^ predominately uses HATS because of low NH_4_^+^ concentrations in the soil^6^.

Currently, only 16 nitrate transporter (NPF) genes are postulated in hexaploid wheat and are localized in all chromosomes except 4A^12^. Expression of these genes is often tissue specific. The genes *NPF6.1* and *NPF6.2* were highly abundant in roots yet low in shoots^12^. *NPF6.3* had similar abundance in the roots and shoots but *NRT6.4* had much higher abundance in the roots. Relative to nitrate transporters, the AMT genes have not been extensively studied in wheat. To date, only two wheat AMT genes – *TaAMT1*;*1* and *TaAMT1;2* – have been reported in the scientific literature^14^.

Uncovering and characterizing the gene sequences of previously undetected AMT (as well as NRT) could help in the study of these genes with relation to N availability and use efficiency (NUE). In crops, NUE is defined as the plants’ capacity to use the applied nitrogen and transform into grains and biomass^15^. N fertilizers can represent a significant cost to farmers and the environment, and as such, optimizing NUE can help reduce the cost of N applications and decrease environmental contamination resulting from excessive N inputs^13^. Understanding how and when nitrate and ammonium transporter genes express in plants from their earliest growth stages is one way to further characterize genotypes with superior NUE. As wheat is one of the most important staple crops in the world, identifying accessions with higher NUE could increase wheat production at lesser cost to meet the demands of a growing global population.

Lately, using next-gen sequencing approaches for crop genomics studies have been gaining popularity. A particular usefulness of the rich next-gen data is that it allows one to obtain an in-depth insight of the genome, including that of the coding and non-coding regions. Sequencing and study of a crop transcriptome can contribute to identification of genes and transcripts involved in important pathways, such as the genes involved in the ammonium and nitrate transport systems identified and discussed in this study. Furthermore, functional annotation of the genes can assist in broadening our knowledge of tissue-specific (as well as generation and stage specific) genes that can further contribute in development of nutrient efficient plant varieties. Our work expands on this idea, and attempts to capture a snapshot of the physiological level landscape of nitrate and ammonium transporter genes in wheat by making use of the publicly available wheat gene sequences^16^. Previously reported ammonium and nitrate transporter studies^12,17–19^ have used fewer genes obtained from less and different plant species. Here, we present a comprehensive set of wheat AMT and NRT genes leveraging on the availability of abundant genomic information and tools.

Therefore, in this study, we present phylogenetic relationships among nitrate and ammonium transporter genes in hexaploid wheat, and their in-seedling expression patterns. A broad list of wheat nitrate and ammonium transporter genes was assembled based on homologous and/or orthologous relationship with genes from the model plant species *Arabidopsis* and four crop species (barley, maize, rice, and wheat). By identifying the molecular pathways involved, we provide putative functional annotation of the assembled genes. The approach taken and methods implemented highlight a simplified process for pathway specific study models in other plant and animal species.

## Results

### Transcriptome sequencing

As the experiment constituted of five wheat lines and two organ types (root and shoot), 10 cDNA libraries were constructed for sequencing. All libraries were paired-end sequenced (100 bp length) on Illumina HiSeq 2500. The sequencing results are summarized in Supplementary Dataset online. The number of raw reads generated from these libraries amounted to 487 million. Removal of reads with Q score <20 resulted in loss of less than 3% of the reads with 483 million reads retained. The average number of high quality clean reads per sample ranged from 42 million to 68 million with an average of 48 million.

### Analysis of differentially expressed genes

For identification of differentially expressed genes (DEGs) between root and shoot of all five lines, sequences from both tissues of each line were aligned separately to the pseudo reference containing 57 wAMT and 500 wNRT gene sequences. As expected, results showed that only a fraction of the captured transcriptome aligned to the reference with <0.05% of reads aligned to wAMT sequences and <0.2% of reads aligned to wNRT sequences (Table 1). For instance, 4,959 and 45,670 reads from Glenlea root samples aligned to wAMT and wNRT sequences, respectively, which is an average of 63–64 reads per gene.

**Table 1:** Read alignment summary of root and shoot transcriptome of five lines to wheat ammonium transporter (wAMT) and wheat nitrate transporters (wNRT) sequences.

Line	Tissue	Filtered read count	Aligned read count		Aligned read %		Average aligned fragment length (bp)	
			wAMT	wNRT	wAMT	wNRT	wAMT	wNRT
Glenlea	Root	47,701,332	4,959	45,670	0.010	0.096	147.73	241.73
Red Fife		67,981,842	4,229	60,970	0.006	0.090	91.33	245.40
Ruby		41,829,496	2510	37,176	0.006	0.089	0.00	233.65
Stoa		43,764,302	3,335	37,681	0.008	0.086	88.17	245.60
Sumai3		41,704,790	2912	42,423	0.007	0.102	77.17	246.99
Glenlea	Shoot	46,455,700	3,461	17,362	0.007	0.037	80.17	251.22
Red Fife		49,665,648	4,063	18,826	0.008	0.038	121.17	252.84
Ruby		49,581,952	3,644	17,528	0.007	0.035	76.67	243.98
Stoa		48,123,910	3,663	19,115	0.008	0.040	134.33	254.01
Sumai3		45,984,938	3,616	16,453	0.008	0.036	88.33	254.37

**Figure 1:**
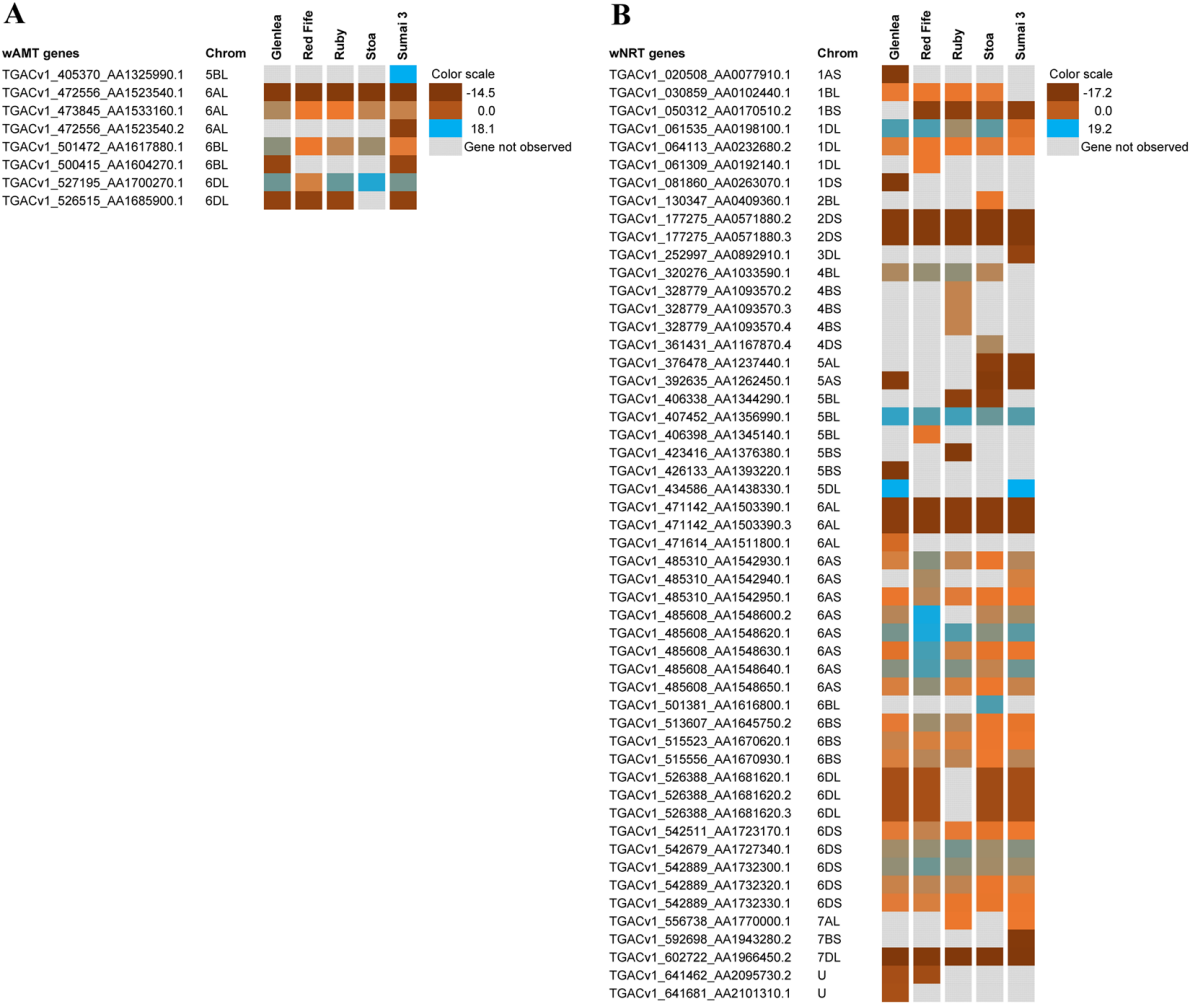
Heatmaps showing intensity (log fold change) of differentially expressed wheat ammonium (wAMT) genes (**A**) and nitrate (wNRT) transporter genes (**B**) in five spring wheat lines. Eight wAMT genes and 52 wNRT genes were found to be differentially expressed between root and shoot of the five lines. Chromosome labeled ‘U’ indicates that the scaffold housing the gene has not been assigned to a chromosome.

Post-alignment, the R packages edgeR and NOISeq were used to statistically compare differential gene expression between root and shoot in all lines. During the analysis in both approaches, an exon was retained only if it was expressed at a count-per-million of >1 in at least 3 samples. This led to reduction of wAMT genes to 53 from 57, and wNRT genes to 459 from 500. An FDR threshold of <0.001 was then applied to obtain the DEGs in edgeR. FDR-correction was not available in NOISeq hence the q value, which is defined as the ratio of probability of differential expression to probability of non-differential expression, was used at 0.9. We provide a comparison between the two methods at the end of this section, yet limit our primary discussion to edgeR results. Eight wAMT DEGs were detected whose expression were significantly different between root and shoot across all five lines. These genes were located on chromosomes 5B, 6A, 6B, and 6D. Specifically, 6 were detected in Glenlea, 5 in Red Fife, 4 in Ruby, 5 in Stoa, and 8 in Sumai3. Of these, four genes were observed in all lines (Fig. 1A). For genes common in ≥2 lines, the log fold change (logFC) in expression was similar for all genes in all lines. Of the 8 wAMT DEGs, four were highly expressed in roots while showing basal or no expression in shoot; and four were highly expressed in shoot while showing none or basal expression in roots (Table 2, Supplementary Dataset). The highly expressed genes in roots are mainly high affinity NH_4_^+^ transporters.

**Table 2:** Annotation information of differentially expressed ammonium transport genes in root and shoot of five wheat seedlings.

wAMT	Transcript ID	Chr: Pos (bp)^a^	ORF (bp)	Amino acid length (bp)	Expression	Gene	GenBank Accession^b^	e value^c^	Reference^d^
wAMT6	TGACv1_473845_AA1533160.1	6AL: 20075–21875	1512	504	higher in root	*OsAMT1*, *AMT1*;*2*	AAB58937.1, AAD17001.1	0	^54^
wAMT7	TGACv1_501472_AA1617880.1	6BL: 9466–11390	1512	504				0	^55^
wAMT8	TGACv1_527195_AA1700270.1	6DL: 18847–20805	1512	504				0	^56^
wAMT17	TGACv1_405370_AA1325990.1	5BL: 3499–6298	1425	475		*AtAMT2*	AAF37192.1, AAM14857.1	0	^57^
wAMT49	TGACv1_472556_AA1523540.1	6AL: 40817–43745	921	307	higher in shoot	ammonium transporter 1-like	AED94172.1, NP_198552.1	1.05E-51	^58^
wAMT50	TGACv1_472556_AA1523540.2	6AL: 40841–43745	897	299				1.65E-51	
wAMT51	TGACv1_500415_AA1604270.1	6BL: 67335–70555	900	300				5.05E-51	
wAMT52	TGACv1_526515_AA1685900.1	6DL: 42967–45945	900	300				2.74E-51	

^a^Chromosome labeled ‘U’ indicates an unknown map position for the scaffold housing the corresponding transcript ID.

^b^Only the two best hits from *blastx* are shown.

^c^The highest value is shown if the *blastx* results had different e-values.

^d^‘NCBI’ is listed as the reference for genes that were either unpublished or directly deposited into NCBI.

**Figure 2:**
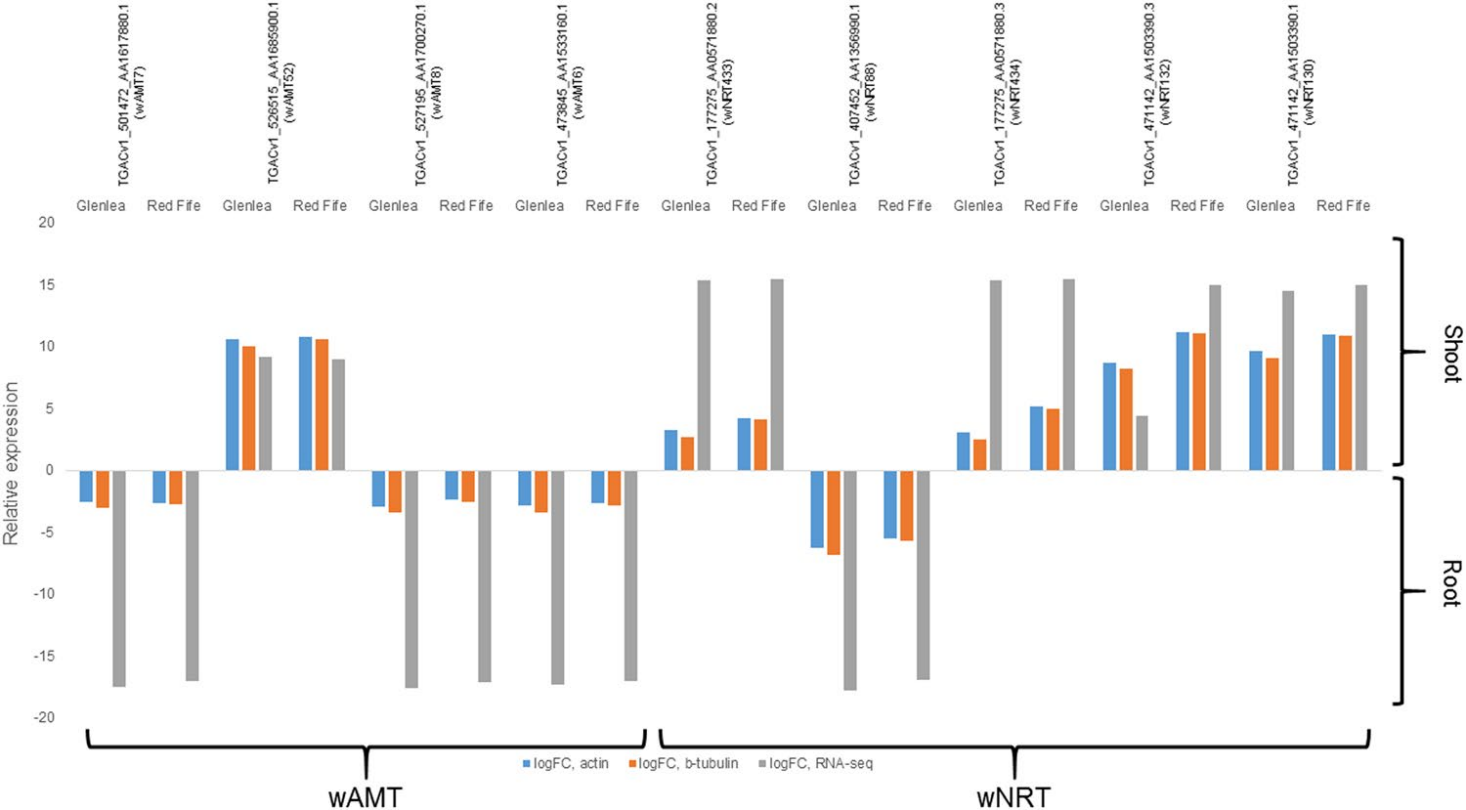
Expression of 9 differentially expressed wheat ammonium (wAMT) and nitrate (wNRT) transporter genes in lines Glenlea and Red Fife. Results from qPCR are means of three biological replicates per line, and are shown in reference to expression of actin and β-tubulin genes; whereas the results from RNA-seq are obtained after normalizing genes against libraries of all five lines. A higher relative expression value in shoot (above X-axis) indicates that the gene was highly expressed in shoot relative to root tissue and a smaller relative expression value in root (below X-axis) indicates that the gene was highly expressed in roots relative to shoot tissue.

For nitrate transporters, 52 wNRT DEGs were detected between root and shoot. The numbers of DEGs within lines were 36 in Glenlea, 33 in Red Fife, 31 in Ruby, 35 in Stoa, and 34 in Sumai3 with 22 common among all lines (Fig. 1B). The wNRT DEGs were distributed in all chromosomes but 2 A, 3 A, and 3B; two genes were not assigned to any chromosome. Of the 52 DEGs, 32 were expressed more in the roots relative to shoot whereas 20 were expressed more in the shoot (Table 3). For genes common in ≥2 lines, logFC values were similar in all lines for all genes except for wNRT15 (*TGACv1_050312_AA0170510.2*), wNRT59 (*TGACv1_061535_AA0198100.1*), and wNRT33 (*TGACv1_485608_AA1548630.1*), where only slight variation among the lines were observed. The wAMT and wNRT DEGs detected by RNA-seq were also validated by qPCR method (Fig. 2). While the magnitude of change differed between qPCR and RNA-seq, all expression trends, i.e. directionality of relative expressions between root and shoot, were confirmed by the qPCR results.

**Table 3:** Annotation information of differentially expressed nitrate transport genes in root and shoot of five wheat seedlings.

wNRT	Transcript ID	Chr: Pos (bp)^a^	ORF (bp)	Amino acid length (bp)	Expression	Gene	GenBank Accession^b^	e value^c^	Reference^d^
wNRT2	TGACv1_485608_AA1548640.1	6AS: 83345–85369	1524	508	higher in root	*NRT2.3*	AAC35883.1, AAC35884.1	0	^59^
wNRT31	TGACv1_485608_AA1548600.2	6AS: 46326–54890	1524	508				0	
wNRT32	TGACv1_485608_AA1548620.1	6AS: 65962–67485	1524	508				0	
wNRT33	TGACv1_485608_AA1548630.1	6AS: 74336–76301	1524	508				0	
wNRT34	TGACv1_485608_AA1548650.1	6AS: 97379–99475	1530	510				0	
wNRT38	TGACv1_320276_AA1033590.1	4BL: 105352–110034	1866	622		*NRT1.2*	AAF07875.1, AAT37840.1	0	NCBI
wNRT49	TGACv1_130347_AA0409360.1	2BL: 11004–15055	1743	581		*NTL1*	AAG51210.1, ABA92279.1	0	^60^
wNRT59	TGACv1_061535_AA0198100.1	1DL: 22846–25580	1791	597		*NPF6.3*	AAK15441.1, AEE28838.1	0	^61^
wNRT64	TGACv1_485310_AA1542930.1	6AS: 98837–100795	1530	510		putative high affinity nitrate transporter	AAK59570.1, AAL07249.1	3.97E-127	NCBI
wNRT69	TGACv1_406398_AA1345140.1	5BL: 48026–52078	1707	569		nitrate transporter	AAM61107.1, BAB02362.1	0	^62^
wNRT76	TGACv1_542679_AA1727340.1	6DS: 50986–52970	1530	510		putative high affinity nitrate transporter	AAN05088.1, AAT66252.1	0	NCBI
wNRT88	TGACv1_407452_AA1356990.1	5BL: 15358–16455	600	200		*NAR2.2*	AAV35211.1, AAP31851.1	≤1.32E-119	^63^
wNRT91	TGACv1_434586_AA1438330.1	5DL: 17026–18225	600	200				≤1.38E-116	
wNRT92	TGACv1_471614_AA1511800.1	6AL: 14946–18203	726	242		*NAR2.1, NAR2.2*	AAV35211.1, AAP31850.1	≤2.31E-110	
wNRT95	TGACv1_501381_AA1616800.1	6BL: 15556–33180	1551	517		*NAR2.1, NAR2.3*	AAP31852.1, AAV35210.1	≤2.31E-73	
wNRT122	TGACv1_328779_AA1093570.2	4BS: 43155–52685	1788	596		putative *NRT1.5*	AAT85061.1, BAC83856.1	0	NCBI
wNRT123	TGACv1_328779_AA1093570.3	4BS: 43155–52685	1788	596				0	
wNRT124	TGACv1_328779_AA1093570.4	4BS: 43155–52685	1788	596				0	
wNRT286	TGACv1_513607_AA1645750.2	6BS: 101176–104615	1935	645		putative low-affinity nitrate transporter	CCJ47268.1, CCJ47217.1	≤3.92E-73	^32^
wNRT383	TGACv1_061309_AA0192140.1	1DL: 134653–137467	1590	530		*NPF5.11*	Q8RX67.1, CCJ47244.1	≤4.69E-119	^61^
wNRT385	TGACv1_361431_AA1167870.4	4DS: 70051–73540	1398	466		*NRT1*	CCJ47237.1	0	^32^
wNRT409	TGACv1_064113_AA0232680.2	1DL: 9517–12105	1812	604		*NPF3.1*	Q9SX20.1, CCJ47253.1	≤1.69E-84	^61^
wNRT410	TGACv1_030859_AA0102440.1	1BL: 75406–78147	1812	604		*NPF3.1*	Q9SX20.1, CCJ47253.1	≤6.22E-83	^61^
wNRT466	TGACv1_556738_AA1770000.1	7AL: 39566–43713	1731	577		nitrate transporter	DAA46638.1, DAA60764.1	0	^64^
wNRT470	TGACv1_485310_AA1542940.1	6AS: 111231–113285	1527	509		*NRT2.3*	Q94JG1.1, XP_015628524.1	2.63E-180	^59^
wNRT471	TGACv1_485310_AA1542950.1	6AS: 120643–122615	1527	509				8.43E-180	
wNRT472	TGACv1_515523_AA1670620.1	6BS: 12762–14655	1524	508				2.83E-180	
wNRT473	TGACv1_515556_AA1670930.1	6BS: 17426–19507	1527	509				7.99E-180	
wNRT474	TGACv1_542511_AA1723170.1	6DS: 140216–142106	1524	508		*NRT2.3; NRT2.5*	Q94JG1.1, XP_008656795.1	0	^59^; NCBI
wNRT475	TGACv1_542889_AA1732300.1	6DS: 5236–7655	1530	510		*NRT2.3*	Q94JG1.1, XP_015628524.1	8.54E-180	^59^
wNRT476	TGACv1_542889_AA1732320.1	6DS: 53472–55365	1527	509				2.63E-180	
wNRT477	TGACv1_542889_AA1732330.1	6DS: 63017–64945	1527	509				1.57E-180	
wNRT12	TGACv1_020508_AA0077910.1	1AS: 21375–23476	1737	579	higher in shoot	putative nitrate transporter	AAB95302.1, AAM20651.1	0	NCBI
wNRT15	TGACv1_050312_AA0170510.2	1BS: 63946–66187	1749	583				0	
wNRT17	TGACv1_081860_AA0263070.1	1DS: 20027–22146	1749	583				0	
wNRT44	TGACv1_602722_AA1966450.2	7DL: 129146–133134	1761	587		*NRT1.2*	AAF07875.1, AAT37840.1	0	
wNRT75	TGACv1_641462_AA2095730.2	U: 72234–75958	1785	595		nitrate transporter	AAM61107.1, BAB02362.1	0	^62,65^
wNRT130	TGACv1_471142_AA1503390.1	6AL: 128730–131585	1698	566		low-affinity nitrate transporter	AAY40798.1, CCJ47257.1	0	^32^; NCBI
wNRT132	TGACv1_471142_AA1503390.3	6AL: 128730–131585	1698	566				0	
wNRT135	TGACv1_526388_AA1681620.1	6DL: 6378–9506	1695	565				0	
wNRT136	TGACv1_526388_AA1681620.2	6DL: 6378–9506	1695	565				0	
wNRT137	TGACv1_526388_AA1681620.3	6DL: 6378–9506	1695	565				0	
wNRT148	TGACv1_423416_AA1376380.1	5BS: 135576–140220	1824	608		*NPF2.9, NPF2.11*	Q9LV10.1, Q9M9V7.1	0	^66,67^
wNRT160	TGACv1_392635_AA1262450.1	5AS: 10736–16703	1851	617		putative *NRT1.5, NPF2.11*	BAC83856.1, Q9LV10.1	0	^66^,NCBI
wNRT230	TGACv1_252997_AA0892910.1	3DL: 7036–10063	1659	553		putative low-affinity nitrate transporter	CCJ47204.1	≤7.79E-30	^32^
wNRT391	TGACv1_592698_AA1943280.2	7BS: 44559–50265	1842	614		*NPF3.1; HvNRT1*	Q9SX20.1, CCJ47238.1	≤3.56E-75	^32^^,^^61^
wNRT395	TGACv1_641681_AA2101310.1	U: 38891–44135	1857	619				≤2.56E-77	
wNRT397	TGACv1_406338_AA1344290.1	5BL: 13904–18310	1755	585		*NPF2.6, NPF2.7*	Q9M1E2.1, Q9M1E1.1	≤2.88E-156	^68^
wNRT433	TGACv1_177275_AA0571880.2	2DS: 153270–156081	1704	568		putative low-affinity nitrate transporter	CCJ47250.1, CCJ47265.1	≤4.91E-50	^32^
wNRT434	TGACv1_177275_AA0571880.3	2DS: 153325–156313	1704	568				≤4.91E-50	
wNRT460	TGACv1_426133_AA1393220.1	5BS: 2–3943	1677	559		low-affinity nitrate transporter	CCN27391.1, CCN27390.1	≤2.22E-84	^12^
wNRT488	TGACv1_376478_AA1237440.1	5AL: 36570–40925	1755	585		*NPF2.3, NPF2.6*	Q9M175.1, Q9M1E1.1	≤2.36E-158	^68^

^a^Chromosome labeled ‘U’ indicates an unknown map position for the scaffold housing the corresponding transcript ID.

^b^Only the two best hits from *blastx* are shown.

^c^The highest value is shown if the *blastx* results had different e-values.

^d^‘NCBI’ is listed as the reference for genes that were either unpublished or directly deposited into NCBI.

Using edgeR and NOISeq we were able to see some differences in results. Five of the 8 wAMT DEGs detected by edgeR were also detected by NOISeq; additionally, NOISeq detected 6 new wAMT DEGs. Similarly, 39 of the 52 wNRT DEGs detected by edgeR were also detected by NOISeq with 17 additional wNRT DEGs. The Pearson’s correlation coefficients (*r*) for the common wAMT and wNRT DEGs between the two methods were 1.0 and 0.99, respectively. Change in logFC estimates among the common wAMT DEGs between the two methods was ±0.6 and that among the wNRT DEGs was ±1.3. The directions of gene regulation were conserved among all common DEGs between the two programs.

### Gene ontology assignment of DEGs

**Figure 3:**
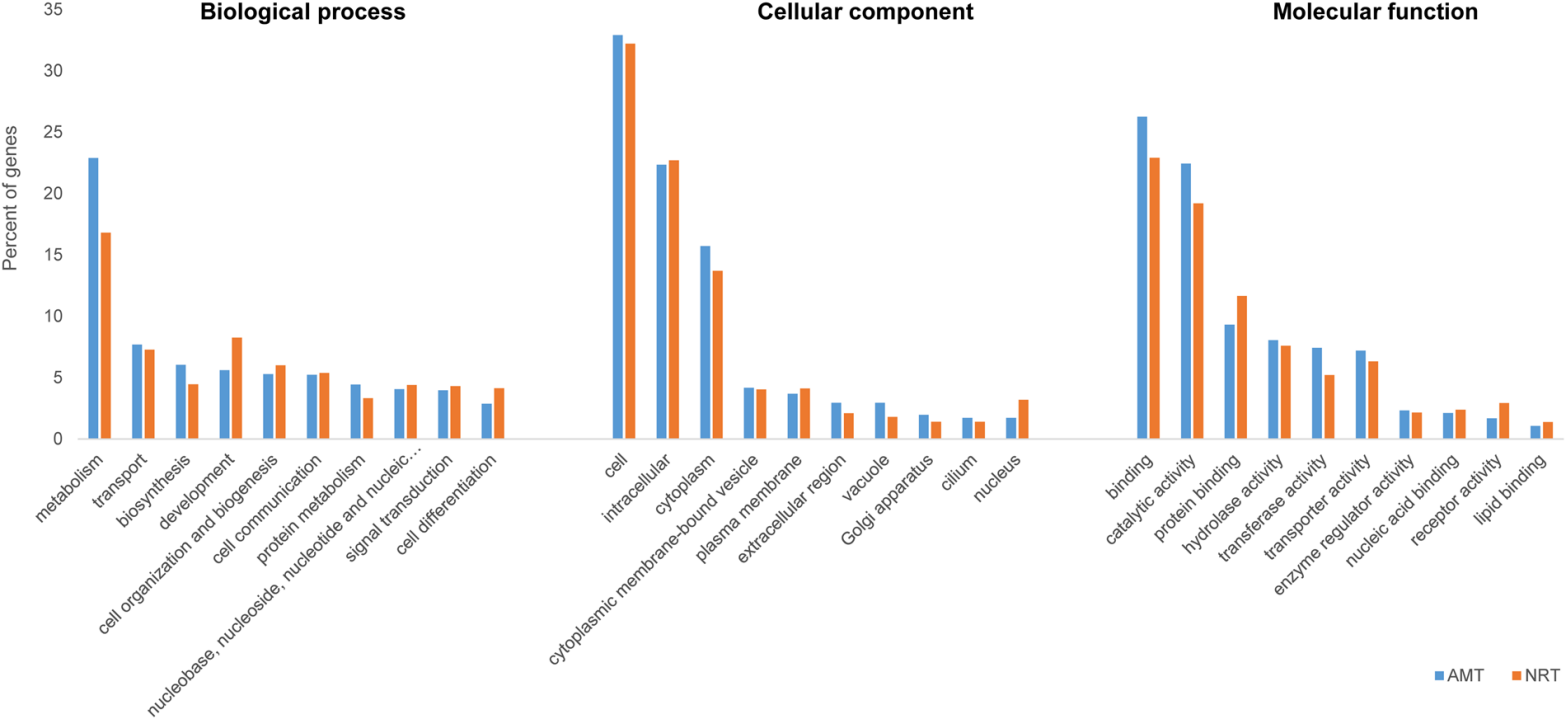
Distribution of differentially expressed wheat ammonium (wAMT) and nitrate (wNRT) transporter genes in three GO categories: biological process, cellular component, and molecular function. Only the top 10 GO terms in each category are displayed.

The total number of wAMT and wNRT DEGs across all five lines were 8 and 52, respectively, with many genes shared among the lines. As this number was relatively small, we combined DEGs from all five lines into one for the purpose of gene ontology (GO) pathway annotation. Results showed the versatility of wAMT and wNRT genes as they were found to be involved in several biological, chemical, and metabolic processes (Fig. 3). These genes were mainly involved in cellular component category, among which their involvement in cell, intracellular, and cytoplasm were considerably higher than in other parts. Functionally, they were mostly involved in binding and catalytic activity with involvement in receptor and transporter activities as well. Under the biological process category, their participation in metabolic process was greater than three-fold compared to other processes such as transport, biosynthesis, and overall cell development.

### Phylogenetic analyses of wheat ammonium and nitrate transporter genes

**Figure 4:**
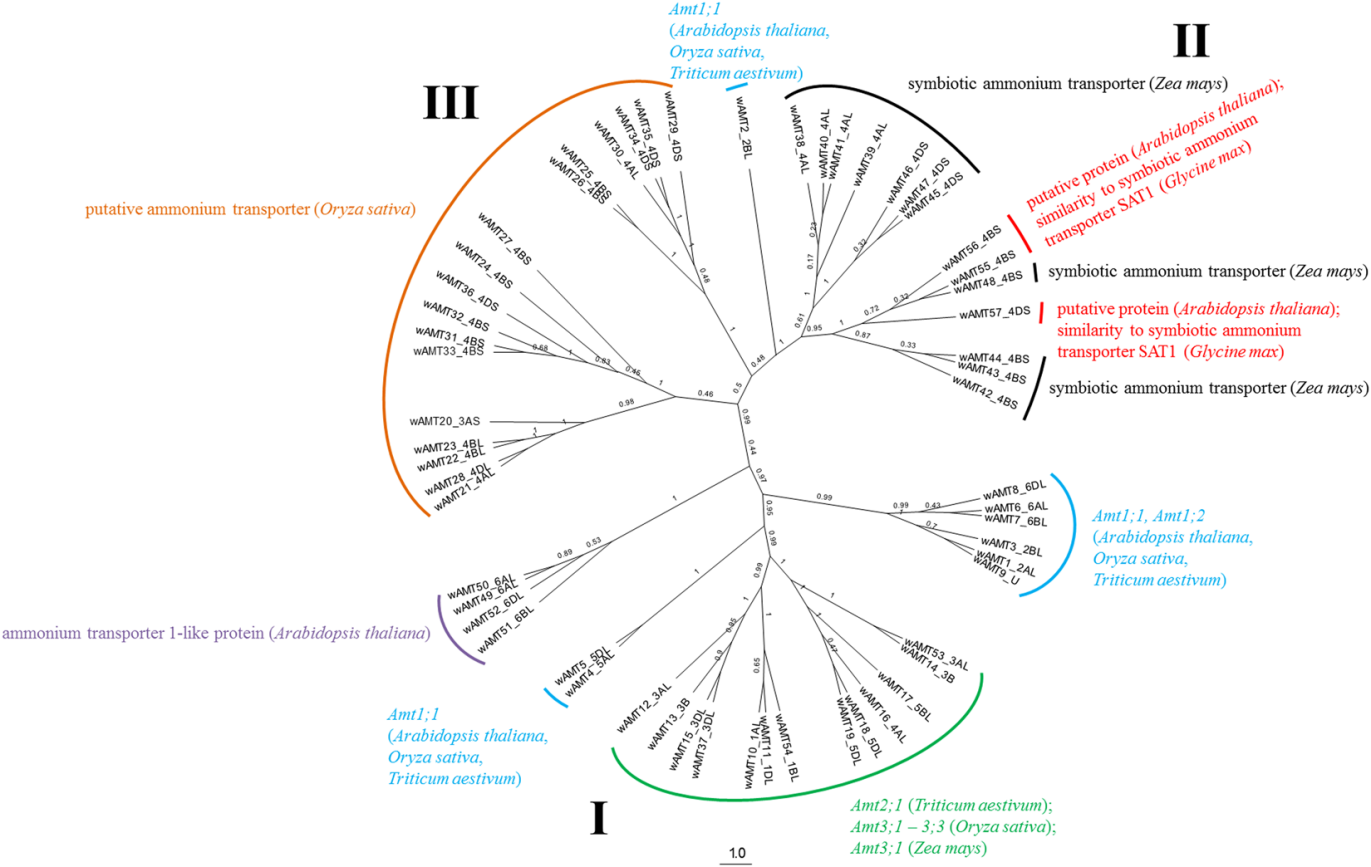
An unrooted Bayesian coalescent tree of wheat ammonium transporter genes constructed from multiple alignment of protein sequences. Posterior probabilities are reported for each branch. Based on the result of *blastx*, the taxa are annotated with either the names of known genes or with GenBank descriptions, and their plant species.

Relationships among several wAMT and wNRT genes were investigated separately using alignment of protein sequences followed by construction of Bayesian trees. Figure 4 shows that the wAMT sequences resolved into three main groups with similar genes or members of same gene families with occasional placements of genes from different gene families into a cluster. In the first group (labeled I), 25 wAMT genes that were highly similar to the *AMT1*, *AMT2*, and *AMT3* genes grouped with each other. The second group (labeled II) formed a distinct cluster consisting of 14 genes annotated as symbiotic ammonium transporters. The third group (labeled III) was of 17 genes similar to *Oryza sativa* accessions that were annotated as putative ammonium transporters.

**Figure 5:**
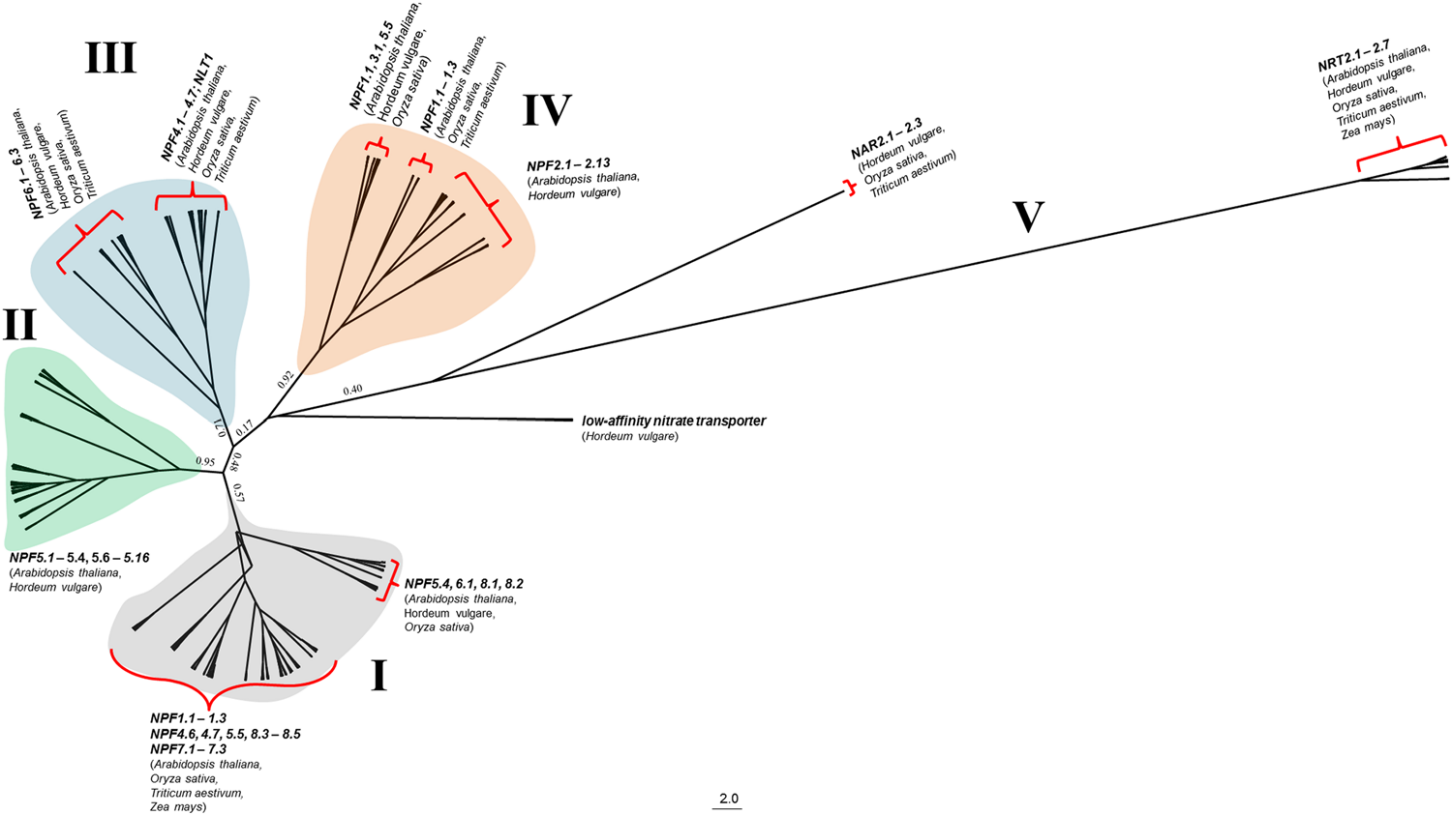
An unrooted Bayesian coalescent tree of wheat nitrate transporter genes constructed from multiple alignment of protein sequences. The clades are divided into five groups, as shown, with Roman numerals I – V. Branches within each clade are annotated with either the names of known genes or with GenBank descriptions, and their plant species, based on *blastx* results. Posterior probabilities are reported only for the main clades. Because of a large taxa number, most of the branch lengths and taxa names are not shown; this information is provided in Supplementary Dataset.

Likewise, the wNRT tree structured into 5 hypothetical clades (Fig. 5). The number of genes were n = 202, 104, 80, 77, and 37 in clades I, II, III, IV, and V, respectively. The wNRT genes in clade I were similar to low affinity nitrate transporter genes (*NPF* groups 1, 4, 5, 7, and 8). Clade II represented wNRT genes with sequence homology to all members of *NPF 5* group except *NPF5.5*. *NPF* groups 4 and 6 comprised most of clade III and *NPF* groups 1 and 2 formed the bulk of clade IV. Lastly, clade V contained wNRT genes with sequence homology to high affinity nitrate transporters of the *NRT2* and *NRT3* (NAR) groups. The clear separation of high affinity genes from the low affinity *NPF* genes indicates evolutionary differences among these gene groups. For both wAMT and wNRT, the posterior probability were quite high with an average of 0.81 (range 0.17–1.00) for wAMT and an average of 0.88 (range 0.02–1.00) for wNRT.

While members of same gene families mostly resolved into close groups, few placements of genes from different gene families into a cluster were also observed (Figs 4, 5). For instance, multiple wNRT genes annotated as (or similar to) *NPF6.1* were found in clades I, III, & V; wNRT genes similar to *NPF1.1*–1.3 and were grouped into clades I and IV; and several wNRT genes annotated as proton-dependent or low-affinity nitrate transporter were grouped in multiple different clades (Supplementary Dataset).

Numerous gene duplications and multiplications were observed among AMT and NRT genes of wheat and other plant species. MEGA7 predicted 8 post-speciation gene duplications among the wheat AMT genes and AMT genes in *Arabidopsis*, maize, and rice; no duplications were observed with barley genes. Similarly, 77 duplications among the wheat NRT genes and NRT genes in *Arabidopsis*, barley, maize, and rice (Supplemental Fig. S2). Several multiplicates of wAMT and wNRT derived from 1–3 genes in *Arabidopsis*, rice and maize with the number of orthologs per multiplicative group often higher than 10. In other words, several wheat genes originated from few genes in *Arabidopsis*, rice, and maize, owing to the homeologous and duplicative nature of the wheat genome^20^. In several instances, wheat genes that were homeologous to each other were found to be duplicates/multiplicates of genes from other plant species. Grouping of these multiplicates supported both wAMT and wNRT phylogenetic trees. For instance, wAMT12–13, wAMT49–52 were orthologous to the *Arabidopsis* ammonium transporter 1-like *AT5G37360* (GenBank accession *AED94172.1*) and form a distinct cluster in Clade I of the wAMT tree (Fig. 4 and Supplemental Fig. S2). Similarly, wAMT20–23 and wAMT28 all are orthologs to the rice putative ammonium transporter *AAP03375.1*, and form a sub-cluster in Clade III. The story was similar in case of wNRTs. Genes wNRT77–86 and wNRT465–467, which are orthologs of three maize genes *DAA60764.1*, *NP_001288684.1*, and *AAO67353.1*, are all placed in Clade I of the wNRT tree (Fig. 5 and Supplemental Fig. S3). Similarly, wNRT118–127 and wNRT384–385 are orthologous to the barley genes *CCJ47236.1*, *CCJ47237.1* and are placed in Clade IV.

### Homeologous genes – phylogenetic analyses & expression

A set of wheat genes were declared homeologous if they all aligned to an AMT or NRT gene with differences (gap and/or mismatch) of ≤2 base pairs in the *blastp* results. Furthermore, the genes had to be present in at least two of the three wheat sub-genomes to be declared homeologous. This led to detection of 8 and 12 sets of homeologous wAMT and wNRT genes, respectively (Supplementary Dataset). While not an exhaustive list, these gene sets provided adequate information to understand their inter-relationships during phylogenetic assignment and in tissue-specific gene expression. Unsurprisingly, the homeologous gene sets often grouped near each other in both wAMT and wNRT trees. For example, three wAMT genes – wAMT6 (*TGACv1_473845_AA1533160.1*), wAMT7 (*TGACv1_501472_AA1617880.1*), and wAMT8 (*TGACv1_527195_AA1700270.1*) – located on chromosomes 6AL, 6BL, and 6DL respectively, were grouped together (Fig. 4). These three genes are most similar with three others – wAMT1, wAMT3, and wAMT9 – of which wAMT1 and wAMT3 are located on 2AL and 2BL respectively whereas wAMT9 has not been assigned to a chromosome. Their placement was not unexpected as all these genes were annotated to the *ammonium transporter 1* (*AMT1*) family. Similarly for wNRT, a group of 16 genes (wNRT40–41, 181, 183, 208, 210–218, 234, and 247) located on chromosomes 4A, 4B, and 4D neighbored each other and belonged to the *nitrate transporter 1* (*NRT1*) family (Fig. 5, Supplementary Dataset). Although most wAMT and wNRT homeolog genes clustered together, not all groupings conformed to this configuration. An example is the placing of wAMT genes 49–52 where wAMT49 and wAMT50 are located on 4DS whereas wAMT51 and wAMT52 are located on 5BL and 5DL, respectively (Fig. 4). Similar observation was made also for wNRT sequences where genes 52–54 belonging to chromosomes 4A, 4B, and 4D were placed closely with genes 46–50 belonging to chromosomes 2A, 2B, and 2D with high posterior probability score (>0.8). Because of the large number of wNRT genes used in drawing the tree, these groupings are not possible to show in the figure; this data is available in Supplementary Dataset.

Expression patterns of homeologous wAMT and wNRT genes in root and shoot tissues of all lines were mostly similar, with few exceptions. Expression levels of wAMT10 (*TGACv1_000306_AA0008460.1*) and wAMT11 (*TGACv1_062093_AA0208670.1*) were significantly different in shoot tissue among the five lines (t-test P value <0.05) whereas no such difference was observed in roots. Similarly, wAMT17 (*TGACv1_405370_AA1325990.1*), located on 5BL, was expressed more in root of all lines whereas wAMT18 (*TGACv1_433945_AA1425980.1*), located on 5DL, was not expressed (Supplementary Dataset). For wheat nitrate transporters, expression of wNRT130–134 (all located on 6A and 6B) was virtually non-existent in roots of all lines relative to that of wNRT135–137 (located on 6D). Expression of wNRT92 (*TGACv1_471614_AA1511800.1*) (located on 6A) was higher in shoot of all lines relative to that of wNRT96 (*TGACv1_502018_AA1622420.1*) (6B) and wNRT97 (*TGACv1_526485_AA1685000.1*) (6D). Expression data for several other homeologous gene sets are available in Supplementary Dataset.

In addition, for both wAMT and wNRT, several instances were observed where expression of a homeolog was high in few lines whereas it was absent in remaining lines. For all wAMT and wNRT genes discussed in the examples above, protein alignments show that several polymorphisms and large insertions-deletions (indels) exist among the genes (see Supplementary Fig. S1).

## Discussion

One of the main goals of this study was to retrieve ammonium and nitrate transporter gene sequences in wheat, and assess their phylogenetic relationships. We observed that such relationships among the wAMT and wNRT genes mostly resulted in grouping of similar genes and gene families.

The number of AMT and NRT genes detected in this study is higher when compared to wheat^12,19^ as well as in other plant species such as *Arabidopsis*, rice, and poplar^11,13,21,22^. One explanation could be the derivation of several copies of wAMT and wNRT genes from a handful of ancestral genes, as seen from duplicated and multiplicated AMT and NRT genes, predicted by MEGA. Specifically, 8 gene duplications were observed among the wheat AMT genes and AMT genes in *Arabidopsis*, maize, and rice; and this number was 77 for the NRT genes (Supplemental Fig. S2). Wheat has a highly repetitive genome derived from inter-species hybridization of three diploid progenitors that occurred <1 million years ago^20,23^. Differences in rates of nucleotide substitution, polyploidization, and overall rates of evolution have been observed in plant species of different ploidy levels, including grasses^24,25^. Therefore, variability in mechanisms used for structural changes in the genome such as gene duplications and inversions are to be expected in a crop with a complex genome such as wheat, as detected by MEGA. Another possible reason is because wheat genome is massively large at approximately 16 giga base pairs and contains over 110,000 genes (ensembl.org), which is more than 2 × the number of genes in rice or poplar. Perhaps the most convincing argument is the fact that wheat has three homeologous sub-genomes which could harbor additional copies of transporter genes, as they have been known to carry multiple copies of several genes^26^. From a technical perspective, the availability of high-quality gene sequences could also have had a significant role in helping us uncover a comprehensive set of AMT and NRT genes in wheat.

As shown in Fig. 4, most AMT genes clustered with each other. Two other groups – putative ammonium transporter and symbiotic ammonium transporter – also formed distinct groups. Often, the resolved groups had genes from the same group of chromosomes. For example, genes belonging to 1A, 1B, 1D or 4A, 4B, 4D were organized among each other, probably owing to higher homeology among the protein sequences of genes in each group of chromosomes. This was also found to be true in the case of nodes with low (<0.2) posterior probability values. As the number of annotated AMT genes in wheat remains small, identification of several AMT genes based on sequence homology to other plant species, of which some are reported here for the first time, should contribute towards identification of even more AMT genes in wheat and their utilization in plant breeding.

Phylogenetic relationships among the NRT genes also showed similar trends as alike genes were grouped together (Fig. 5). The NRT genes are mainly divided into three categories: ‘*NRT1* (recently named as *NPF* (*NRT1/ PTR FAMILY*)), *NRT2* (‘high-affinity’ genes), and *NRT3* (recently named as *NAR*). We annotated our Bayesian phylogenetic tree with 5 clades of which clade V, the most evolutionarily distant clade, constituted of high-affinity *NRT2* and *NRT3* (*NAR2*) wheat genes. The remaining clades constituted of low-affinity transporters from *NRT1* family - which have been now designated as *NPFX.Y* in the new nomenclature^16^- and mostly resolved into several smaller groups based on the gene families. As seen in Figs 4 and 5, some known genes overlap across different clades (example: *NPF6.1*). This was indeed true for both wAMT and wNRT genes where distribution of non-similar genes among multiple branches was observed. One explanation for this is that the protein coding motifs of different wAMT/wNRT genes are similar enough to represent the same gene during *blastx* search. Another explanation is that a gene being positioned in different clades simply suggests that the wAMT/wNRT sequences with which they are associated with could have different sequences and therefore are different from each other; yet based on the *blastx* hits, depending on the similarity %, are still the same. Exploring the relationships among ammonium and nitrate transporters of different species have been reported before, and is not new to wheat^12,21,22,27,28^. Hence, the close associations of wAMT and wNRT genes with ammonium and nitrate uptake genes in other plant species shown in our work are expected to yield similar functions in wheat.

We also used root and shoot transcriptome from five wheat lines – Glenlea, Red Fife, Ruby, Stoa, and Sumai3 in order to uncover differentially expressed AMT and NRT genes between root and shoot during early growth stages of wheat. We used two R packages – edgeR and NOISeq for DEG detection. Majority of the genes we found using edgeR were also confirmed by NOISeq results, and *vice-versa*. The residual differences in DEG detection between the two packages are to be expected because they follow different approaches during DEG analysis. The biggest point of contrast between the two is their underlying assumption of transcript abundance: edgeR assumes a negative binomial distribution of the read counts whereas NOISeq adapts to the data on a nonparametric method. Another difference we observed was how genes with 0 reads or count per million (cpm) reads were handled. In edgeR, the cpm values are taken and processed as-is for DEG detection whereas NOISeq replaces genes with cpm = 0 with a user-determined value. These led to differences in detection as well as in lack of detection of some genes during the comparative analysis. Choice of the appropriate program is therefore a function of number of available biological and technical replicates, handling of missing data, and the presumed read distribution. Depending on the dataset used for analysis, both methods are known to show varying results in terms of number of genes detected, false discoveries, and precision^29,30^.

Across all lines, 8 wAMT genes were differentially expressed of which four were common in all lines (Fig. 1). Interestingly, 7 of the 8 DEGs were located in group 6 chromosomes and one was located on 5B. It is also worth noting that all 8 wAMT genes were located on long arms of their respective chromosomes. It has been previously shown that *TaAMT1;1* is located on 2BL^31^ yet the authors report that this gene could also belong to 2AL, 2BL, or 6DL. In our study, the wAMT gene *TGACv1_527195_AA1700270.1* was located on 6D and found to be identical with *TaAMT1;1* (GenBank accession *AAS19466.2*) with full-length alignment of 503 bp and e-value of 0 (Supplementary Dataset). Therefore, we can predict with high confidence that one copy of *TaAMT1;1* is definitively located on 6DL of wheat. As previously discussed, the wheat AMT genes are poorly studied and the genomic locations of all wheat AMTs are unknown. In this study, we have uncovered 57 wheat AMT genes. We hope that our discovery adds to the annotation effort of the wheat transcriptome. Simultaneously, using these reported sequences in validation of wAMT genes could help understand their functions and pave the path to use them in breeding.

The 52 differentially expressed wNRT genes were identified in all chromosomes except in 2A, 3A, and 3B. Buchner and Hawkesford^12^ have reported the presence of the seven NPF families in all 21 wheat chromosomes. In particular, chromosome 2A contains *TaNPF2.3* and *TaNPF6.7*; and both 3A and 3B contain *TaNPF1.1*, *TaNPF2.4*, and *TaNPF2.5*. As these genes were detected during *blastx*-search (Supplementary Dataset, Fig. 5), it is possible that they were not differentially expressed between root and shoot tissues in the lines used in our experiment. We found that *TaNRT2* (GenBank accession *AAG01172.1*) was highly similar with 10 genes located in chromosomes 6A, 6B, and 6D of which five genes, all located on 6AS, were differentially expressed (Fig. 1, Fig. 5); Buchner and Hawkesford (2014) did not observe any transporter family 2 genes on group 6 chromosomes. A handful of genes, including low-affinity nitrate transporter genes, that were initially detected in tissues of adult barley plants by Kohl *et al*.,^32^, were also observed in our wheat seedlings. As the involvement of several nitrate transporters in early plant growth and development has already been established^33–35^, these findings confirm that N-transport genes are very important to the wheat plant and are differentially expressed between root and shoot during its early stages. Also, our discovery suggests that not all nitrate transporter genes may be expressed in a wheat plant during its early stages.

Tissue-specific expression of several wAMT and wNRT DEGs were congruent with previous studies (Table 2). For example, *TGACv1_473845_AA1533160.1* (wAMT6) was highly expressed in the roots of all five lines, and is highly matches the ammonium transporter gene *AMT1;2* in *Arabidopsis thaliana* (GenBank accession *AAD38253.1*). *AMT1;2* is primarily expressed in the endodermal and cortical cells of roots and carries out the uptake of high affinity ammonium^36^. Another example was the *HvPTR1*-like low-affinity nitrate transporter gene (GenBank accession *CCJ47250.1*) reported by Kohl *et al*.,^32^ in barley shoot tissue (flag leaf, glumes, and grains) during seed development. This gene was similar to *TGACv1_177275_AA0571880.2* (wNRT433) on chromosome 2D and was found to be expressed highly in shoot tissue of all five lines. This indicates that this gene is mostly active in the shoot and is involved in acquiring nitrate from the roots.

Several wAMT and wNRT genes also exhibited differential expression patterns in previously published studies, which we uncovered by aligning these genes with wheat RNA-seq transcripts from those studies^37–39^ (Supplementary Dataset). For instance, the wNRT genes *TGACv1_392635_AA1262450.1* (*AtNRT1.5*) decreased in transcript abundance post-anthesis flag leaf whereas genes *TGACv1_471614_AA1511800.2* (*AtNAR2.2*) and *TGACv1_423416_AA1376380.1* (*AtNPF2.11*) increased in abundance on post-anthesis flag leaf. Likewise, the wAMT genes *TGACv1_096146_AA0317430.1*, *TGACv1_129977_AA0400880.1*, and *TGACv1_129977_AA0400870.1* (all similar to *OsAMT1*) increased in expression levels post-anthesis on flag leaf whereas *TGACv1_472556_AA1523540.1*, *TGACv1_472556_AA1523540.2*, and *TGACv1_500415_AA1604270.1* had higher expression levels during anthesis. We mostly observed that the overall expression of the wAMT and wNRT genes in these studies did not follow a strict pattern, implying that expression is a function of genotype as well as conditional differences. Nearly all the genes expressed in these datasets were also present in our study, suggestive of a comprehensive compilation of wheat AMT and NRT transcripts in wheat seedlings despite being a single-point capture. Although we discovered these genes in 21-days-old wheat seedlings, their detection in previously published datasets that studied adult wheat plants emphasizes their importance in all plant growth stages.

Lastly, we would also like to highlight few genes that were undetected during DEG analysis (largely due to filtering parameters) but had interesting expression patterns. Seventeen wNRT genes in 11 chromosomes were not expressed in either root or shoot of the five lines; 9 of these 17 genes are annotated as putative low-affinity nitrate transporters (Supplementary Dataset). Forty-two genes did not express in roots of any of the five lines, of which three were identified as DEGs in shoot of all five lines. Similarly, 72 genes were completely absent in shoot. Of these 72, 17 were identified as DEGs in the roots of which 15 were highly expressed in all five lines. In case of wAMT genes, we did not find genes that were not expressed in the roots. However, 14 wAMT genes were not expressed in shoot of any line of which three were identified as DEGs (Supplementary Dataset). Additionally, several sets of homeologous genes exhibited differential expression that were generally tissue-specific. The genes in these sets contained several polymorphisms and indels among their alignments (Supplementary Fig. S1). While the differences among the homeologous sequence pairs might be rendering each gene different function relative to others, a definitive statement cannot be made with the amount of data we have. Our group is currently conducting a follow-up study to understand involvement of genes when different N levels are supplied to the wheat plant in controlled environment as well as in the field. Upon completion of this project, we hope to uncover the relationship among these tissue-specific non-expressed and highly expressed genes in wheat seedlings with adult wheat plants.

## Methods

### Creation of a ‘pseudo reference’ containing wheat AMT and NRT genes

One of the main objectives of this study was to assemble the hexaploid wheat complement of ammonium transporter (AMT) and nitrate transporter (NRT) genes. To this end, protein sequences of AMT and NRT genes of *Arabidopsis* (*Arabidopsis thaliana*), barley (*Hordeum vulgare*), maize (*Zea mays*), rice (*Oryza sativa*), and wheat (*Triticum aestivum*) were obtained by nominal title search on NCBI. Initially, 164 AMT sequences ranging in length 186–533 bp and 312 NRT genes ranging in length 3–660 bp were obtained. Upon removal of duplicates (those with 100% sequence similarity), 46 AMT and 235 NRT unique sequences remained. These sequences were BLAST-searched against the *Triticum aestivum* TGACv1 (build 34, ENSEMBL) coding sequences (CDS) in order to create a ‘pseudo reference’ which only includes wheat AMT and NRT sequences. This task was carried out using the *blastp* command in NCBI’s local blast program (version 2.5.0 +). To allow for possible identification of gene homeologs in the three wheat sub-genomes, top 10 alignment results with e-value <1E-10 for each AMT and NRT genes were retained. Thus obtained wheat CDS were parsed to remove duplicate gene IDs. The final pseudo reference consisted of 57 wheat AMT genes and 500 wheat NRT genes. These two groups are referred to as wAMT and wNRT in the text, respectively. Inter-species gene duplications were predicted using MEGA7^40^, which implements the method of Zmasek and Eddy^41^ to identify duplicates based on speciation. All sequences described above are available in Supplementary Dataset.

### Assessment of phylogenetic relationship

Phylogenetic analyses for wAMT (57 sequences) and wNRT (500 sequences) genes were performed separately using the Bayesian approach in BEAST 1.8.4^42^. First, protein sequences of wAMTs and wNRTs were aligned separately in MUSCLE 3.8.31^43^ using 100,000 iterations. To identify the most appropriate evolutionary model, the multiple protein alignment generated by MUSCLE was passed to ProtTest3 3.4.2^44^. ProtTest3 results showed that the best model for wAMT alignment was the JTT (Johns-Taylor-Thornton) model^45^ with gamma-distributed rate variation (G); whereas the best model for wNRT alignment was JTT with gamma and invariant (I) sites. So the models JTT+ G and JTT + G + I were used for wAMT and wNRT, respectively, in BEAUti to generate input files for BEAST. For both models, a relaxed clock with an uncorrelated log-normal model with a constant size coalescent tree prior was chosen. Tree construction was initiated with a random tree for each alignment, and was carried out for 10,000,000 generations with sampling of one tree in every 1000 generations. The program Tracer 1.6.0 was used to analyze the BEAST output, and the first 25% trees from each run were determined as burn-in and discarded from the analysis. The resulting wAMT and wNRT trees were drawn in FigTree 1.4.3.

### Plant material and experimental design

Five spring wheat lines – ‘Glenlea’ (CItr 17272), ‘Red Fife’ (CItr 6196), ‘Ruby’ (CItr 6047), ‘Stoa’ (PI 520297), ‘Su Mai No. 3’ (PI 481542; referred as Sumai3 in the manuscript) – were obtained from the National Small Grains Collection (NSGC) in Aberdeen, ID, USA. These lines were originally selected for a different, ongoing study by the same authors, from which a subsection of the produced RNA-seq data was utilized for this study. Briefly, all lines were grown in a growth chamber with constant temperature of 20° Celsius, 12h-12h day-night cycle, and relative humidity of approximately 60%. Seedlings were grown in sand and were irrigated with tap water until they developed two full leaves (Zadoks scale 12; approximately 9 days)^46^. From the 10^th^ day, they were treated with a modified Hoagland’s solution with the following chemical composition: 6.0 mM KNO_3_, 4.0 mM Ca(NO_3_)_2_.4H_2_O, 0.5 mM NH_4_H_2_PO_4_, 2.0 mM MgSO_4_.7H_2_O, 25 μM H_3_BO_3_, 0.5 μM MnCl_2_.4H_2_O, 0.5 μM ZnSO_4_.7H_2_O, 0.2 μM (NH_4_)_6_Mo_7_O_24_.4H_2_O, 0.5 μM CuSO_4_.5H_2_O, and 45 μM FeCl_3_. Each line was grown in three replications with each replicate having three seedlings. When the seedlings entered tillering stage (21 days old, Zadoks scale 22–25), roots and shoots of each replicate with three seedlings were harvested separately for RNA extraction and whole transcriptome sequencing. Tillering stage was chosen given the positive correlation of tillering with N supply^47,48^. Tissues for RNA extraction were flash-frozen in liquid N_2_ and stored in −80° C until RNA extraction.

### Whole transcriptome sequencing

Total RNA from root and shoot tissue was extracted using the Trizol reagent (Fisher Scientific, Carlsbad, CA). The RNA was treated with DNaseI (Promega, Madison, WI), cleaned using QIAquick PCR Purification Kit (Qiagen, Germantown, MD), and checked for quality and quantity using BioAnalyzer 2100 (Agilent Technologies, Santa Clara, CA). For each line, equal amount of RNA from three replicates of root tissues were pooled together and three replicates of shoot tissues were pooled together to create cDNA libraries. The libraries were created following Illumina’s instructions, multiplexed (10 samples in one lane), and sequenced on Illumina’s HiSeq 2500 using the 100 bp paired-end protocol. Obtained sequences were filtered for minimum phred quality score (Q) of 20 followed by adapter and barcode trimmed using Trimmomatic 0.36, and assigned to the corresponding sample.

### Differential gene expression analysis

Indexing of both wAMT and wNRT genes was conducted using the *index* command in kallisto 0.43.0^49^. Alignment of the sequenced paired end transcripts to wAMT and wNRT genes was also done in kallisto (*quant* command) with 100 bootstrapping of the input reads. The resulting alignment counts were further analyzed for differentially expressed genes (DEGs) using the edgeR^50^ package in R 3.3.1 using a false discovery rate (FDR) of <0.001. A second R package, NOISeq, was used with recommended threshold (q) of 0.9 to cross validate the results from edgeR. NOISeq is a nonparametric approach to identify differentially expressed genes in samples without technical replications^51^. For DEG analysis in both methods, in-root expression was compared with in-shoot expression within each line. Assigning definite physiological functions to the DEGs on an individual basis is a difficult task and is outside the scope of our work. Therefore, a holistic gene ontology (GO) analysis was carried out to obtain a snapshot of the general functions of these genes. DEGs were categorized into GO slim categories using the method of Hu *et al*.,^52^.

### qRT-PCR validation of DEGs

RNA-seq results were further evaluated by performing qPCR on 9 DEGs that were expressed highly in either root or in shoot tissues. Because we saw similar expression trends in the RNA-seq results across all five lines, only Glenlea and Red Fife were used in qRT-PCR. First-strand cDNA was synthesized using 1 µg of total RNA using TaqMan Reverse Transcription Reagents (ThermoFisher, Waltham, MA). Expression of genes was quantified using iTaq Universal SYBR Green Supermix (Bio-Rad, Hercules, CA) on the CFX Connect Real-Time PCR Detection System (Bio-Rad). The analysis was carried out using three biological replicates per tissue per line. For internal control, the housekeeping genes Actin and β-tubulin were used^53^. Primer sequences for all genes are available in Supplementary Dataset.

### Data availability

All sequences generated during the experiment have been uploaded to NCBI’s short read archive under bioproject PRJNA397654 (sample accessions: SAMN07501955 – SAMN07501974). All other pertinent data generated or analyzed during this study are included in the Supplementary Information files.

## Electronic supplementary material

Supplementary Dataset

Supplementary Figures
