# Supplementary material for: Phylogenetic analyses and in-seedling expression of ammonium and nitrate transporters in wheat
Source: Sci Rep. 2018 May 4;8:7082. doi: 10.1038/s41598-018-25430-8 (PMC5935732; doi:10.1038/s41598-018-25430-8)

Phylogenetic analyses and in seedling expression of ammonium and nitrate transporters in wheat

Prabin Bajgain[1, 2], Blake Russell[1], Mohsen Mohammadi[1*]

1 Department of Agronomy, Purdue University, West Lafayette, IN 47907, USA

2 Department of Agronomy and Plant Genetics, University of Minnesota, St. Paul, MN 55108, USA

* mohamm20@purdue.edu

Supplementary Figure S1: Alignment of protein sequences for two sets each of homeologous wAMT and wNRT genes. Genes within each alignment showed different expression pattern in either tissue (root or shoot) relative to other genes. Gray color represents similar amino acids.

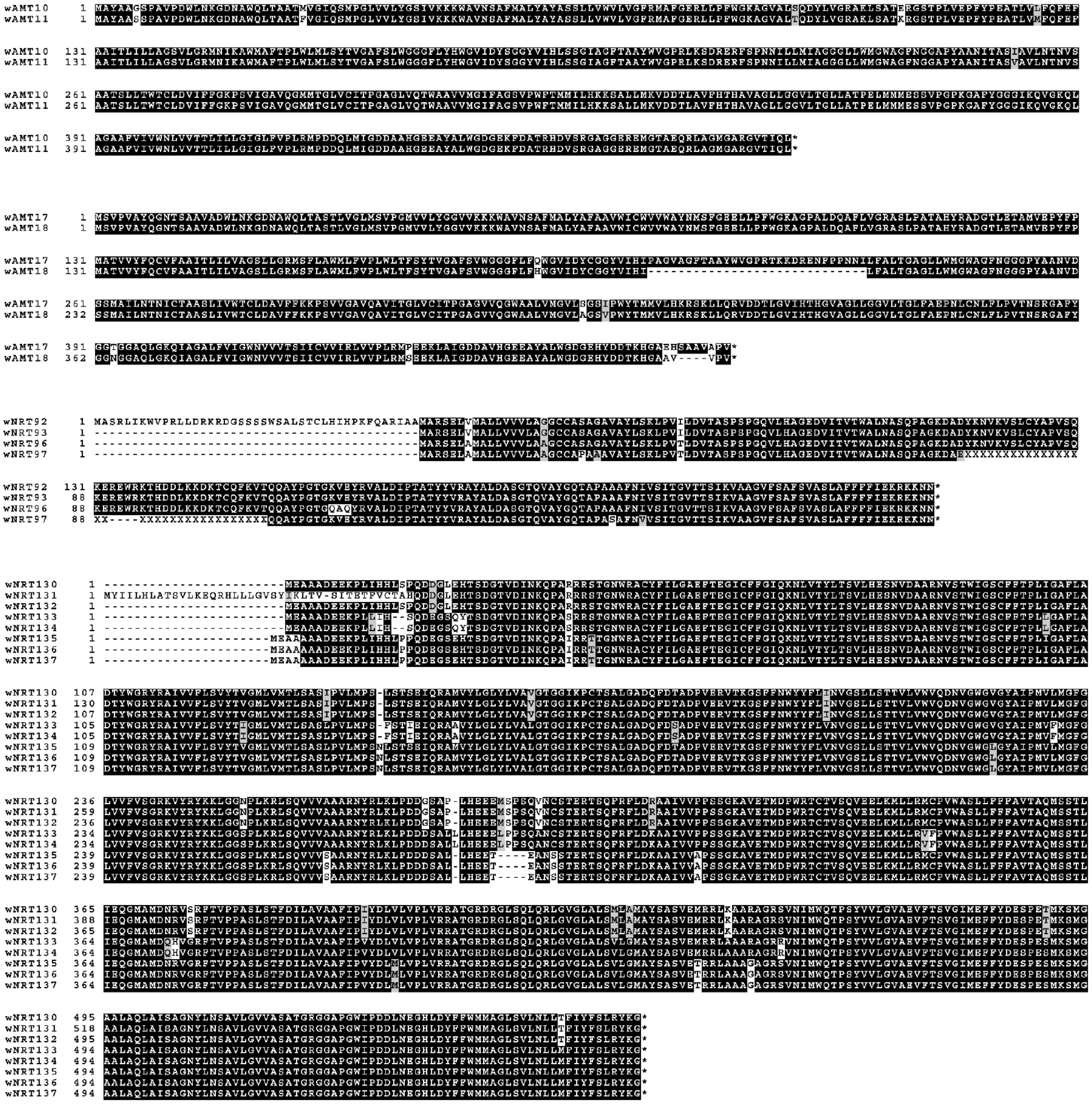

Supplementary Figure S2:
Evolutionary relationships among ammonium transporter (AMT) genes in 5 plant species - Arabidopsis, barley, maize, rice, and wheat; Duplications are labeled with open diamonds

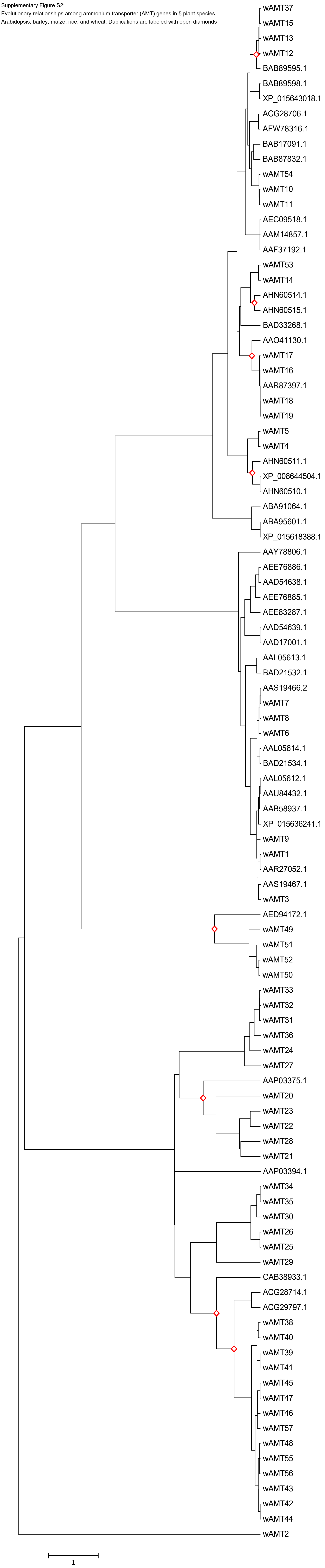

Supplementary Figure S3:
Evolutionary relationships among nitrate transporter (NRT) genes in 5 plant species - Arabidopsis, barley, maize, rice, and wheat; Duplications are labeled with open diamonds

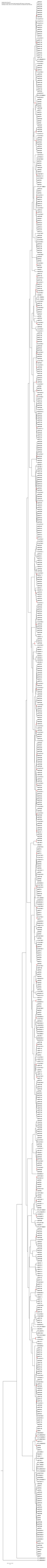

Supplement: Supplementary file 2 — Supplementary Figures [file 41598_2018_25430_MOESM2_ESM.pdf]
